# Evaluation for clinical benefit of metformin in patients with idiopathic pulmonary fibrosis and type 2 diabetes mellitus: a national claims-based cohort analysis

**DOI:** 10.1186/s12931-022-02001-0

**Published:** 2022-04-11

**Authors:** Taylor T. Teague, Stephanie R. Payne, Bryan T. Kelly, Timothy M. Dempsey, Rozalina G. McCoy, Lindsey R. Sangaralingham, Andrew H. Limper

**Affiliations:** 1 Division of Pulmonary and Critical Care Medicine, Mayo Clinic, 200 First Street SW, Rochester, MN 55905 USA; 2 Mayo Clinic Robert D. and Patricia E. Kern Center for the Science of Health Care Delivery, Harwick Building, Second Floor, 200 First Street SW, Rochester, MN 55905 USA; 3 OptumLabs, Eden Prairie, MN USA; 4 Division of Community Internal Medicine, Mayo Clinic, 200 First Street SW, Rochester, MN 55905 USA; 5 Department of Pulmonary and Critical Care Medicine, Mayo Clinic, 200 First Street SW, Rochester, MN 55905 USA

**Keywords:** Idiopathic pulmonary fibrosis, Interstitial lung disease, Metformin, Type 2 diabetes mellitus, Mortality, Hospitalization

## Abstract

**Background:**

Idiopathic pulmonary fibrosis (IPF) is a chronic progressive lung disease with high morbidity and limited treatment options. Type 2 diabetes mellitus (T2DM) is a common comorbid illness among patients with IPF and is often treated with metformin, the first-line agent in the management of T2DM. There is growing evidence demonstrating metformin’s anti-fibrotic properties; however, there is little real-world clinical data regarding its potential effectiveness in IPF. This study aims to evaluate the clinical benefit of metformin in patients with IPF and T2DM.

**Methods:**

This nationwide cohort study used de-identified administrative claims data from OptumLabs® Data Warehouse to identify 3599 adults with IPF and concomitant T2DM between January 1, 2014 and June 30, 2019. Two cohorts were created: a cohort treated with metformin (n = 1377) and a cohort not treated with metformin (n = 2222). A final 1:1 propensity score-matched cohort compared 1100 patients with IPF and T2DM receiving metformin to those with both diagnoses but not receiving metformin; matching accounted for age, sex, race/ethnicity, residence region, year, medications, oxygen use, smoking status, healthcare use, and comorbidities. Outcomes were all-cause mortality (primary) and hospitalizations (secondary).

**Results:**

Among 2200 patients with IPF and T2DM included in this matched analysis, metformin therapy was associated with a reduction in all-cause mortality (hazard ratio [HR], 0.46; 95% confidence interval [CI], 0.36–0.58; *p* < 0.001) and hospitalizations (HR, 0.82; 95% CI, 0.72–0.93; *p* = 0.003) compared to patients not receiving metformin.

**Conclusions:**

Among patients with IPF and T2DM, metformin therapy may be associated with improved clinical outcomes. However, further investigation with randomized clinical trials is necessary prior to metformin’s broad implementation in the clinical management of IPF.

## Background

Idiopathic pulmonary fibrosis (IPF) is a chronic progressive interstitial lung disease with high morbidity, mortality, and limited treatment options [1, 2]. Many therapies have been investigated as potential treatment options for IPF, but most have been ineffective [3–7] and, in some cases, harmful [8–10]. Currently, only two medications are approved by the U.S. Food and Drug Administration (FDA) for IPF: the anti-fibrotic agents nintedanib and pirfenidone, which were approved in 2014 based on phase 3 clinical trials demonstrating slowed decline in lung function in patients with IPF [11, 12]. Subsequent pooled analyses [13–16] and observational studies [17, 18] suggested that their use reduces the risk of hospitalizations and improves mortality. Yet, only between 25 and 60% of patients with IPF are prescribed these anti-fibrotic medications [19–22]. Many factors contribute to their limited use including high costs (estimated at $100,000 per year) [23], side effects, uncertainty regarding IPF diagnosis, and treatment deferral for presumed stable disease [19, 24]. Thus, additional therapies are needed to improve the health outcomes and reduce the risk of death among patients with IPF.

Type 2 diabetes mellitus (T2DM) is a common chronic health condition that is present in many patients with IPF [17]. Metformin is the first-line glucose-lowering medication in the management of T2DM [25]. In addition to metformin’s anti-glycemic control, there is accumulating evidence demonstrating anti-neoplastic [26–30], anti-aging [31–34], and anti-fibrotic properties [35–40]. Regarding its potential anti-fibrotic effect, initial in vivo studies showed that metformin reduced TGFβ1-induced fibrosis in human bronchial fibroblasts [41]. Several subsequent in vitro studies in murine models of bleomycin-induced lung fibrosis showed that mice treated with metformin after bleomycin exposure had decreased profibrotic markers [37, 38] as well as reduced histological [35–40] and radiological [40] signs of lung fibrosis compared to mice not receiving metformin. While these laboratory findings are intriguing, the concentrations and relative doses used in the cellular and rodent models were quite high, raising questions as to whether metformin would have similar impact on fibrosis in people with T2DM and IPF.

Beyond these pre-clinical findings, there is a paucity of clinical data regarding metformin’s effectiveness in patients with IPF. In fact, current evidence is limited to two studies. The first is a single post hoc analysis of three phase 3 clinical trials of pirfenidone where Spagnolo et al. investigated the effect of metformin on the clinical benefit of patients with IPF [42]. Metformin did not significantly impact clinical outcomes, including forced vital capacity (FVC) decline, 6-min walking distance (6MWD) decline, and death. However, this study was limited in its applicability and interpretation given the very small number of patients with metformin use (n = 71; 11.4%) and lack of formal power analysis. The second study is a retrospective study where Lambert et al. evaluated for significance of cardiovascular drugs on disease progression and survival in patients with idiopathic pulmonary fibrosis [43]. Metformin was again found to have no significant impact on IPF progression (demonstrated by annual FVC and diffusing capacity of the lungs for carbon monoxide (DLCO) decline) or survival. However, this study was also limited by its very small number of patients receiving metformin (n = 28).

Given the overall rarity of IPF, we chose to utilize a large healthcare claims database to enhance patient population diversity and size. To date, this study represents the only observational cohort analysis that evaluates for clinical benefit of metformin in patients with IPF in real-world practice. This nationwide cohort study sought to compare the risk of all-cause mortality among patients with IPF and concomitant T2DM receiving metformin to those not receiving metformin. In secondary analysis, the risk of hospitalizations was compared between IPF patients with and without metformin use.

## Methods

### Data source

We conducted a retrospective cohort study of de-identified administrative claims data from OptumLabs® Data Warehouse (OLDW), which is a large U.S. healthcare database containing information of enrollees in private and Medicare Advantage health plans [44]. Enrollees differ in age, race, ethnicity, incomes, and geographic location with representation from all 50 states. Per the Health Insurance Portability and Accountability Act of 1996 [45], institutional review board approval was not required since completely de-identified patient data was used.

### Study population

The study population consisted of adults (≥ 18 years old) with IPF included in OLDW between January 1, 2014, and June 30, 2019. The diagnosis of IPF was established using either *International Classification of Diseases*, 9th edition (ICD-9) or *International Classification of Diseases*, 10th edition (ICD-10) codes for IPF or claims for ant-fibrotic medications present in medical and pharmacy claims, respectively. Patients were required to have either a single inpatient claim or two outpatient claims for IPF, as previously detailed by Dempsey et al. [17]. All patients were required to have 6 months of continuous enrollment in the health plan. The index date was defined as the first claims evidence of IPF after the 6-months enrollment criterion was met. Patients were also required to have a diagnosis of T2DM which was established using validated Healthcare Effectiveness Data and Information Set (HEDIS) criteria (an ICD-9 or ICD-10 billing code for T2DM, the use of insulin or oral anti-hyperglycemic agent, and either a single inpatient claim or two outpatient claims for T2DM) [46]. We excluded patients with invalid demographic data and type 1 diabetes mellitus (T1DM), defined as an ICD-9 or ICD-10 billing code for T1DM.

### Comparator groups

**Fig. 1:**
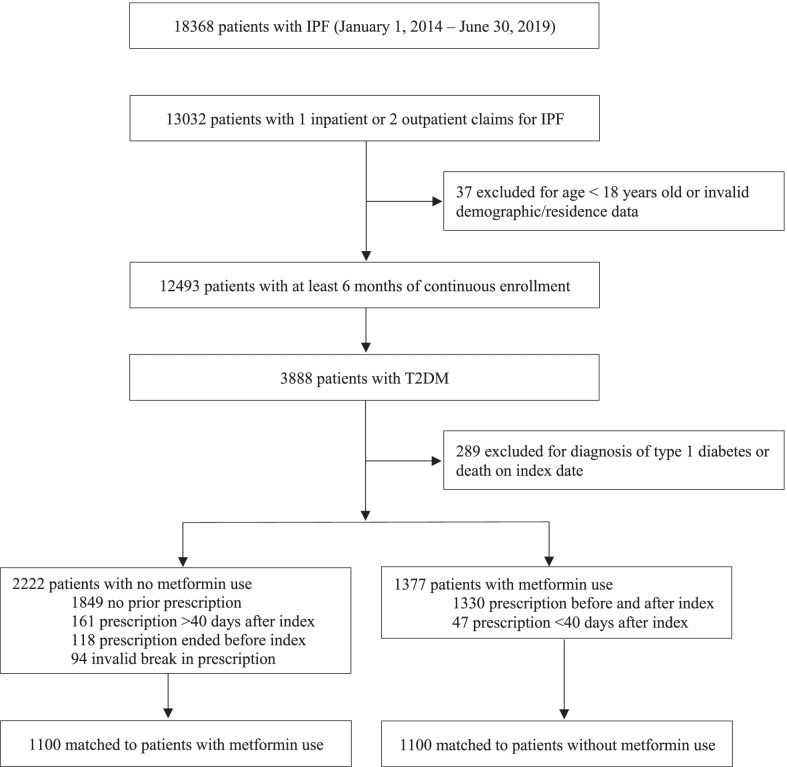
Flow diagram of OptumsLab® cohort creation. *IPF* idiopathic pulmonary fibrosis, *T2DM* Type 2 diabetes mellitus, *T1DM* Type 1 diabetes mellitus

Patients with IPF were divided into those treated with metformin and those not treated with metformin, as seen in Fig. 1. The metformin-treated group included patients with a claim for metformin (any dose) any time prior to the index date and up until 40 days after the index date (to account for patients who started metformin after their index date). A 40-day window period was chosen because it was the median number of days (interquartile range 17, 78) to first metformin fill after index date. Most patients in the metformin-treated group had pharmacy claims for metformin that preceded their index date (1330/1377; 96.6%). Patients whose metformin prescription began > 40 days after the index date were placed in the comparator group and censored at time of treatment initiation (161/2222; 7.2%). The comparator group included those who had no prior prescription for metformin (1849/2222; 83.2%), those who discontinued metformin prior to index date (118/2222; 5.3%), and those who had an invalid break in prescription (94/2222; 4.2%).

Patients were then matched 1:1 on age, sex, race, residence region, year, medications (see “Independent variables” section), oxygen use, smoking status, healthcare use (see “Independent variables” section), and comorbidities (cardiac arrhythmia, congestive heart failure, chronic pulmonary disease, depression, hypertension, obesity, pulmonary circulation disorder, renal failure, rheumatoid arthritis, and valvular disease). As in our previous studies, we used oxygen use as a measure for disease severity matching since lung functions measures are not available in this dataset [17].

### Independent variables

Comorbidities diagnosed in the 6 months prior to index date were identified using ICD-9 and ICD-10 diagnostic codes associated with medical claims. Comorbidity burden was evaluated using the Elixhauser sum of conditions [47]. Healthcare use including hospitalizations, primary care office visits, and pulmonologist office visits were captured by medical claims in the 6 months preceding the index date. Medication use (specifically oral corticosteroids, angiotensin-converting enzyme (ACE) inhibitors, angiotensin receptor blockers (ARBs), statins, sodium-glucose co-transporter-2 (SGLT2) inhibitors, insulin, dipeptidyl peptidase-4 (DPP4) inhibitors, glucagon-like peptide-1 (GLP-1) receptor agonists, sulfonylureas, glitazones, and anti-fibrotics) was defined as having filled a prescription within 6 months prior to the index date. Oxygen use was identified using Healthcare Common Procedure Coding System indicating oxygen supplies during the 6 months prior to index date.

### Follow up

Patients were followed until the end of the study period (June 30, 2019), end of enrollment in health insurance plan, death, start of first prescription fill for metformin (for untreated cohort) or metformin discontinuation (for treated cohort) which was defined as no prescription fill 30 days after the end supply.

### Study outcomes

Primary outcome was all-cause mortality. Secondary outcome was all-cause hospitalizations.

### Statistical analysis

We used propensity score matching to balance the differences in baseline characteristics between those receiving metformin and those not receiving metformin. A propensity score was estimated using logistic regression based on age, sex, race, residence region, year, medications (see “Independent variables” section), oxygen use, smoking status, healthcare use (see “Independent variables” section), and comorbidities (cardiac arrhythmia, congestive heart failure, chronic pulmonary disease, depression, hypertension, obesity, pulmonary circulation disorder, renal failure, rheumatoid arthritis, and valvular disease). Specifically, we used one-to-one nearest-neighbor caliper matching to match patients based on the logit of the propensity score [48]. We evaluated the standardized difference to assess the balance of covariates after matching, and a standardized difference ≤ 10% was considered acceptable [49]. When balance was not achieved through propensity score matching, we controlled for the unbalanced variable in the analysis. We then used Cox proportional hazard regression to compare mortality and hospitalizations in the matched cohort between patients on metformin and those not on metformin.

Falsification endpoint analysis was performed with one endpoint selected: fracture. Corresponding ICD-9 and ICD-10 diagnostic codes are listed in Table 1.

**Table 1:** Diagnosis codes

Diagnosis	ICD-9	ICD-10
Idiopathic pulmonary fibrosis	516.31	J84.112
Diabetes mellitus, type 2	250. × 0, 250. × 2	E11.xxx, O24.1xx
Diabetes mellitus, type 1	250. × 1, 250. × 3	E10.xxx
Fracture	733.1x, 733.8x, 733.93, 733.94, 733.95, 733.96, 733.97, 733.98, 800–829	M48.4x, M80.8x, M81.8, M83.3x-6x, S02.x, S12.x, S22.x, S32.x, S42.x, S52.x, S62.x, S72.x, S82.x, S92.x

*ICD-9* International Classification of Diseases, 9th edition; *ICD-10* International Classification of Diseases, 10th edition

For a sensitivity analysis, we used inverse probability treatment weighting (IPTW) instead of propensity score matching to repeat the main analysis. A weight of 1/propensity score was used for patients receiving metformin and 1/(1-propensity score) for those not receiving metformin.

All analyses were conducted using SAS 9.4 (SAS Institute Inc.) and Stata version 15.1 (StataCorp).

## Results

### Characteristics of the patient population

A total of 18,368 patients were examined for eligibility. There were 3599 (19.6%) patients with IPF and concomitant T2DM who met eligibility criteria and were included in the study. A total of 1377 (38.2%) were treated with metformin upon cohort entry. Of these 1377 patients, 1100 were 1:1 propensity score matched to patients with IPF and T2DM that had not received metformin, as seen in Fig. 1. The baseline clinical characteristics were well balanced between the groups with standardized differences < 10%, as shown under Table 2.

**Table 2:** Baseline characteristics of patients before and after propensity score matching

	No. (%) of patients							
	Before propensity score matching				After propensity score matching			
	No metformin (n = 2222)	Metformin (n = 1377)	Std diff	*p* value	No metformin (n = 1100)	Metformin (n = 1100)	Std diff	*p* value
Age, years				< 0.001				0.88
Mean	73.4 (9.4)	72.2 (8.5)	–		72.4 (9.4)	72.5 (8.8)	–	
Median (IQR)	75 (68–82)	73 (67–78)	–		73 (67–80)	74 (68–79)	–	
Age group, years				< 0.001				0.63
18–54	89 (4.0)	45 (3.3)	0.0%		48 (4.4)	40 (3.6)	0.0%	
55–64	278 (12.5)	187 (13.6)	3.2%		155 (14.1)	146 (13.3)	0.0%	
65–74	729 (32.8)	572 (41.5)	18.1%		419 (38.1)	411 (37.4)	0.6%	
75 +	1126 (50.7)	573 (41.6)	0.0%		478 (43.5)	503 (45.7)	0.0%	
Sex				0.02				0.73
Female	1042 (46.9)	591 (42.9)	0.0%		511 (46.5)	503 (45.7)	2.0%	
Male	1180 (53.1)	786 (57.1)	8.0%		589 (53.5)	597 (54.3)	0.0%	
Race				0.004				0.99
White	1332 (59.9)	794 (57.7)	7.9%		645 (58.6)	647 (58.8)	0.0%	
Black	334 (15.0)	170 (12.3)	0.0%		158 (14.4)	151 (13.7)	0.0%	
Hispanic	358 (16.1)	263 (19.1)	7.9%		191 (17.4)	193 (17.5)	0.0%	
Asian	64 (2.9)	60 (4.4)	2.1%		35 (3.2)	34 (3.1)	0.2%	
Unknown	134 (6.0)	90 (6.5)	0.0%		71 (6.5)	75 (6.8)	0.8%	
Insurance				0.85				0.32
Commercial	245 (11.0)	149 (10.8)	–		135 (12.3)	120 (10.9)	–	
Medicare advantage	1977 (89.0)	1228 (89.2)	–		965 (87.7)	980 (89.1)	–	
Census Region				0.54				0.71
Midwest	535 (24.1)	324 (23.5)	0.0%		243 (22.1)	264 (24.0)	0.0%	
Northeast	336 (15.1)	200 (14.5)	0.0%		166 (15.1)	156 (14.2)	3.0%	
South	1167 (52.5)	720 (52.3)	0.0%		600 (54.5)	586 (53.3)	0.9%	
West	184 (8.3)	133 (9.7)	4.8%		91 (8.3)	94 (8.5)	0.0%	
Year				0.60				1.00
2014	392 (17.6)	224 (16.3)	0.0%		187 (17.0)	187 (17.0)	2.0%	
2015	349 (15.7)	204 (14.8)	0.0%		168 (15.3)	171 (15.5)	0.0%	
2016	416 (18.7)	254 (18.4)	0.0%		217 (19.7)	210 (19.1)	1.7%	
2017	466 (21.0)	288 (20.9)	0.0%		229 (20.8)	229 (20.8)	0.0%	
2018	409 (18.4)	273 (19.8)	3.6%		202 (18.4)	206 (18.7)	0.0%	
2019*	190 (8.6)	134 (9.7)	4.1%		97 (8.8)	97 (8.8)	0.0%	
Anti-fibrotics	401 (18.0)	391 (28.4)	24.7%	< 0.001	260 (23.6)	255 (23.2)	0.0%	0.80
Baseline medications								
Steroids	1009 (45.4)	600 (43.6)	0.0%	0.28	505 (45.9)	484 (44.0)	2.0%	0.37
ACE inhibitor	530 (23.9)	469 (34.1)	22.6%	< 0.001	344 (31.3)	330 (30.0)	1.7%	0.52
ARB	558 (25.1)	433 (31.4)	14.1%	< 0.001	331 (30.1)	322 (29.3)	0.1%	0.67
Statins	1296 (58.3)	1005 (73.0)	31.2%	< 0.001	756 (68.7)	739 (67.2	0.0%	0.44
SGLT2	28 (1.3)	50 (3.6)	–	< 0.001	13 (1.2)	29 (2.6)	–	0.01
Insulin	664 (29.9)	248 (18.0)	0.0%	< 0.001	231 (21.0)	232 (21.1)	0.0%	0.96
DPP4	229 (10.3)	176 (12.8)	–	0.02	143 (13.0)	120 (10.9)	–	0.13
GLP-1	50 (2.3)	63 (4.6)	–	0.00	33 (3.0)	42 (3.8)	–	0.29
Sulfonylureas	483 (21.7)	426 (30.9)	21.0%	< 0.001	294 (26.7)	294 (26.7)	0.0%	1.0
Glitazones	58 (2.6)	71 (5.2)	–	0.00	38 (3.5)	51 (4.6)	–	0.16
HbA1c lab result				0.00				0.04
Mean (SD)	6.9 (1.3)	7.0 (1.2)	–		6.9 (1.3)	7.0 (1.2)	–	
Median (IQR)	6.6 (6.1–7.5)	6.8 (6.2–7.5)	–		6.6 (6.1–7.4)	6.8 (6.2–7.5)	–	
Primary care office visit	1821 (82.0)	1209 (87.8)	–	< 0.001	939 (85.4)	964 (87.6)	–	0.12
Pulmonary office visit	1237 (55.7)	843 (61.2)	11.3%	0.00	652 (59.3)	659 (59.9)	0.7%	0.76
Baseline hospitalizations				< 0.001				0.92
0	1192 (53.6)	925 (67.2)	27.9%		693 (63.0)	701 (63.7)	0.0%	
1	636 (28.6)	326 (23.7)	0.0%		280 (25.5)	277 (25.2)	0.0%	
2 +	394 (17.7)	126 (9.2)	0.0%		127 (11.5)	122 (11.1)	0.8%	
DME, oxygen	1001 (45.0)	587 (42.6)	0.0%	0.16	479 (43.5)	473 (43.0)	0.4%	0.80
Current smoker	646 (29.1)	395 (28.7)	0.0%	0.80	342 (31.1)	321 (29.2)	0.2%	0.33
Elixhauser comorbidity count				< 0.001				0.93
Mean (SD)	6.9 (3.1)	5.8 (2.7)	–		6.1 (2.7)	6.1 (2.8)	–	
Median (IQR)	7 (5–9)	6 (4–7)	–		6 (4–8)	6 (4–8)	–	
Elixhauser conditions								
Cardiac arrhythmia	905 (40.7)	405 (29.4)	0.0%	< 0.001	357 (32.5)	364 (33.1)	1.3%	0.75
Congestive heart failure	941 (42.3)	417 (30.3)	0.0%	< 0.001	371 (33.7)	376 (34.2)	4.3%	0.82
Chronic pulmonary disease	1525 (68.6)	895 (65.0)	0.0%	0.02	737 (67.0)	722 (65.6)	0.0%	0.50
Depression	368 (16.6)	176 (12.8)	0.0%	0.00	160 (14.5)	156 (14.2)	0.0%	0.81
Hypertension	1828 (82.3)	1130 (82.1)	0.0%	0.88	916 (83.3)	908 (82.5)	1.4%	0.65
Obesity	371 (16.7)	227 (16.5)	0.0%	0.87	186 (16.9)	183 (16.6)	1.8%	0.86
Pulmonary circulation disorder	512 (23.0)	300 (21.8)	0.0%	0.38	250 (22.7)	248 (22.5)	1.9%	0.92
Renal failure	764 (34.4)	206 (15.0)	0.0%	< 0.001	186 (16.9)	203 (18.5)	0.0%	0.34
Rheumatoid arthritis	300 (13.5)	167 (12.1)	0.0%	0.23	148 (13.5)	143 (13.0)	1.3%	0.75
Valvular disease	575 (25.9)	276 (20.0)	0.0%	0.00	239 (21.7)	238 (21.6)	0.0%	0.96

*SGLT2* sodium-glucose co-transporter-2, *DPP4* dipeptidyl peptidase-4, *GLP-1* glucagon-like peptide-1, *DME* durable medical equipment

*From January 1,2019–June 30, 2019

The mean ages of metformin-treated and untreated cohorts were 72.5 years (SD, 8.8) and 72.4 years (SD, 9.4), respectively. Men comprised 53.5% of the cohort untreated with metformin and 54.3% of the metformin-treated cohort. In the overall unmatched cohort, the percentage of patients with IPF that received an anti-fibrotic medication was 18.0% in the metformin-untreated cohort and 28.4% in the metformin-treated group. After 1:1 matching, the percentage of patients with IPF that received an anti-fibrotic medication was 23.6% in the metformin-untreated cohort and 23.2% in the metformin-treated group. The mean HbA1c was 6.9 (SD, 1.3) for the metformin-untreated cohort and 7.0 (SD, 1.2) for the metformin-treated cohort. The most prevalent comorbidities in the overall unmatched cohort were hypertension (82.2%), other chronic pulmonary conditions (67.2%), and congestive heart failure (37.7%). Mean durations of observation were 292.4 days (SD, 316.3) and 432.2 days (SD, 431.2) in the treated and untreated cohorts, respectively.

### Association of metformin use with all-cause mortality

**Fig. 2:**
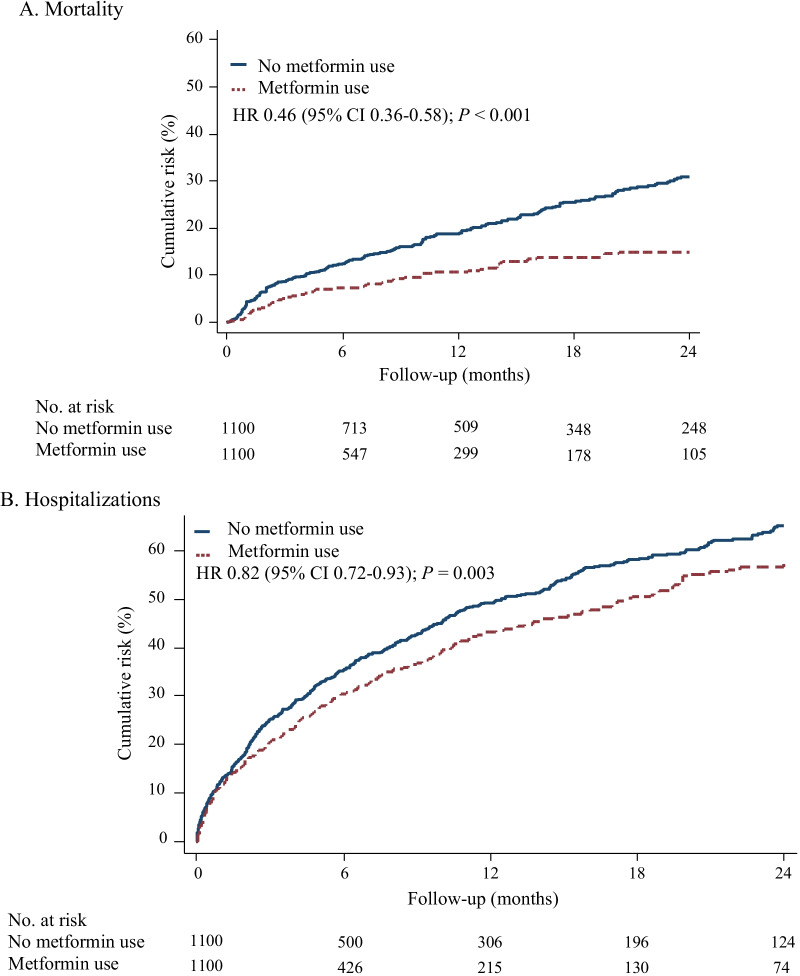
Kaplan–Meier curves displaying cumulative risk of mortality (**A**) and hospitalizations (**B**) among patients with idiopathic pulmonary fibrosis with and without metformin use

We next analyzed whether the use of metformin in patients with IPF and T2DM was associated with different all-cause mortality in the study population. Metformin use in the cohort diagnosed with both IPF and T2DM was associated with a significant decreased risk of all-cause mortality (hazard ratio [HR], 0.46; 95% confidence interval [CI], 0.36–0.58; *p* < 0.001) compared to patients with both diagnoses that were not receiving metformin, as shown in Fig. 2A and Table 3.

**Table 3:** Cox proportional hazard regression comparing mortality and hospitalizations among patients with idiopathic pulmonary fibrosis with and without metformin use

		Rate per 100 (95% CI)				
	Before propensity score matching		After propensity score matching		Hazard ratio (95% CI)	*p* value
	No metformin (n = 2222)	Metformin (n = 1377)	No metformin (n = 1100)	Metformin (n = 1100)		
Death	25.1 (23.3, 27.1)	10.5 (8.7, 12.5)	20.6 (18.4, 23.2)	10.5 (8.5, 12.8)	0.46 (0.36, 0.58)	< 0.001
Hospitalization	76.1 (72.0, 80.6)	55.12 (50.4, 60.3)	64.3 (59.2, 69.9)	57.8 (52.4, 63.8)	0.82 (0.72, 0.93)	0.003

### Association of metformin use with hospitalizations

In a similar manner, we further sought to determine whether the use of metformin in patients with IPF and T2DM was associated with differing rates of hospitalization. Metformin use in this cohort was associated with a significant decreased risk of hospitalizations (HR, 0.82; 95% CI, 0.72–0.93; *p* = 0.003), as demonstrated in Fig. 2B and Table 3.

### Falsification analysis and sensitivity analysis

We then performed falsification endpoint analysis to test for confounding. These results are depicted in Table 4 and confirm our findings (HR, 0.75; 95% CI, 0.45–1.21; *p* = 0.23). We also performed a sensitivity analysis using IPTW as seen in Table 5 which demonstrated similar results to our main analysis.

**Table 4:** Falsification endpoint analysis

		Rate per 100 (95% CI)				
	Before propensity score matching		After propensity score matching		Hazard ratio (95% CI)	*p* value
	No metformin (n = 2222)	Metformin (n = 1100)	No metformin (n = 2222)	Metformin (n = 1100)		
Fracture	4.57 (3.81, 5.48)	2.54 (1.75, 3.68)	4.00 (3.05, 5.25)	3.01 (2.05, 4.4)	0.75 (0.45, 1.21)	0.23

**Table 5:** Sensitivity analysis using inverse probably treatment weighting

	Before propensity score matching		After propensity score matching		Hazard ratio (95% CI)	*p* value
	No metformin (n = 2222)	Metformin (n = 1377)	No metformin (n = 2222)	Metformin (n = 1377)		
Death	25.1 (23.3, 27.1)	10.5 (8.7, 12.5)	22.5 (20.7, 24.6)	11.6 (9.2, 14.7)	0.46 (0.36, 0.58)	< 0.001
Hospitalization	76.1 (72.0, 80.6)	55.12 (50.4, 60.3)	70.8 (65.7, 76.3)	63.4 (55.7, 72.3)	0.81 (0.72, 0.92)	0.001

### Effects of cardiovascular disease and renal disease on treatment effects of metformin in IPF with T2DM

Finally, we assessed the potential differences in metformin effect based upon whether patients had coded cardiovascular and renal diseases including arrythmia, congestive heart failure (CHF), peripheral vascular disease (PVD) or renal disease. We did not observe any significant differences in the effects of metformin based on the presence or absence of these other coded diagnoses (Table 6).

**Table 6:** Effects of cardiovascular and renal disease on treatment effects of metformin in IPF with T2DM

	Matched		Treated adjusted hazard ratio [95% CI]	p-value for interaction
	No metformin (N = 1100)	Metformin (N = 1100)		
Arrhythmia				0.41
Yes	30.64 (25.52, 36.78)	17.07 (12.70, 22.94)	0.49 (0.34, 0.69)	
No	16.80 (14.41, 19.57)	7.67 (5.76, 10.21)	0.42 (0.31, 0.60)	
CHF				0.07
Yes	30.96 (26.05, 36.79)	21.52 (16.21, 28.55)	0.58 (0.41, 0.81)	
No	16.04 (13.67, 18.83)	6.64 (4.93, 8.96)	0.39 (0.28, 0.55)	
PVD				0.39
Yes	24.12 (18.95, 30.71)	15.00 (10.05, 22.37)	0.56 (0.35, 0.90)	
No	19.76 (17.27, 22.59)	9.43 (7.42, 11.98)	0.44 (0.33, 0.58)	
Renal				0.52
Yes	25.12 (19.42, 32.50)	15.19 (9.90, 23.29)	0.55 (0.33, 0.90)	
No	19.72 (17.28, 22.49)	7.32 (7.56, 12.08)	0.44 (0.34, 0.58)	

## Discussion

Metformin therapy in patients with IPF and concomitant T2DM was observed to have a significant 54% reduction in all-cause mortality in this large nationwide claims-based dataset. This is striking and represents a far stronger effect than previously observed with anti-fibrotic therapy in a similar population, where anti-fibrotic agents reduced the risk of all-cause mortality by 23% [17]. Metformin was further associated with an 18% lower risk of hospitalization for any cause. In a similar population, the two FDA-approved anti-fibrotic medications only had a trend towards decreased hospitalization [18].

The anti-fibrotic medications nintedanib and pirfenidone are currently the only FDA approved medications with demonstrated clinical benefit in IPF, yet they are underutilized with these medications being prescribed in only about 25% to 60% of patients with IPF in various studies [19–22]. Their use is limited due to high cost (estimated in the U.S. at $100,000 per year) [23], adverse side effects, uncertainties regarding the diagnosis of IPF, and treatment deferral for presumed stable disease [19, 24]. In contrast however, metformin is affordable, widely available, and safe. It serves as the first-line agent in the management of T2DM [25] and is used to treat select patients with pre-diabetes [50] as well as an alternative agent in patients with gestational diabetes [51] and polycystic ovarian syndrome [52, 53].

There are laboratory investigations that provide some insights into the possible effectiveness of metformin in IPF. As previously mentioned, metformin has been shown to reduce lung fibrosis in murine models of bleomycin-induced lung fibrosis [35–40]. While the mechanism by which metformin reduces the development of lung fibrosis and accelerates its resolution is unknown, several hypotheses exist. The predominant hypothesis revolves around metformin-mediated stimulation of adenosine monophosphate-activated protein kinase (AMPK) [36, 38], which is a critical cellular energy sensor and regulator of cellular metabolism [54, 55]. AMPK protects cellular functions by converting cells to a catabolic state after metabolic stressors (i.e. hypoxia) interfere with adenosine triphosphate production [56, 57]. However other metformin-mediated signaling pathways have been implicated in the reduction of lung fibrosis including suppression of the pro-fibrotic cytokine insulin-like growth factor-1 (IGF-1) [40] and activation of BMP2-PPAR (bone morphogenetic protein-2-peroxisome proliferator-activated receptor) gamma that ultimately results in trans-differentiation of myofibroblasts to lipofibroblasts [39].

It is important to emphasize that our current study only observes an association of metformin use with better clinical outcomes in patients with both IPF and T2DM and does not establish causality nor provide any information as to whether this agent has altered the course of the fibrotic disease itself. Claims-based analyses such as this cannot assess the impact of metformin on surrogate endpoints of fibrotic disease progression, like FVC decline, DLCO decline, or 6-min walk test. Instead, the current study provides significant initial insights into the clinically meaningful and patient-centric endpoints of mortality and hospitalizations. Indeed, metformin may reduce mortality and hospitalizations through its effects on cardiovascular disease, respiratory complications of lung fibrosis, or overall health of the patient. Alternatively, perhaps there is an interaction between the anti-fibrotic medications and metformin that potentiates each other’s effect—hence heightening the vast differences in observed outcomes. Metformin may offer cardiovascular protection to patients with IPF as it does in patients with T2DM [58–61]. In that light, it should be noted that cardiac disease is a common cause of death in patients with IPF, second only to respiratory failure [62, 63].

This study has several limitations. First, ICD billing codes were used to identify patients with IPF. IPF is difficult to diagnose and often requires multidisciplinary review for accurate diagnosis [64], so relying solely on billing codes for identification may result in inclusion of patients with erroneous diagnoses. However, we used the most accurate billing codes for IPF available for our review [65], a method that has been used previously in other studies [17, 22]. In addition, given the observational nature of this analysis, confounding factors cannot be completely eliminated, despite our rigorous statistical adjustments, falsification testing, and propensity score-matching. Our third limitation is due to cohort design as our treated cohort consisted of patients who filled a prescription for metformin. Unfortunately, in such a real-world analysis it is not possible to guarantee all patients reliably took the medication. Regardless, patients in our treated cohort consistently filled their prescriptions and those that did not refill metformin were excluded.

## Conclusions

To our knowledge, this is the first real-world study assessing the clinical benefit of metformin in patients with IPF and T2DM. Several clinically relevant observations were gleaned from this U.S national, claims-based retrospective cohort analysis, most importantly that metformin therapy in patients with IPF and T2DM was associated with a significant reduction in all-cause mortality. While these results are intriguing, caution is encouraged. Specific recommendations for the clinical management of idiopathic pulmonary fibrosis will require further investigation, including randomized clinical trials that ideally delineate between cardiovascular protection, disease stabilization, and improvement in the course of fibrosis by utilizing objective measures such as pulmonary function testing and thoracic imaging.

## Data Availability

The data underlying the results of this study are third party data owned by OptumLabs and contain sensitive patient information; therefore, the data is only available upon request. Interested researchers engaged in HIPAA compliant research may contact connected@optum.com for data access requests. The data use requires researchers to pay for rights to use and access the data.

## Abbreviations

IPF: Idiopathic pulmonary fibrosis
T2DM: Type 2 diabetes mellitus
TGFβ1: Transforming growth factor-beta 1
HR: Hazard ratio
CI: Confidence interval
U.S.: United States
FDA: Federal Drug Administration
FVC: Forced vital capacity
6MWD: 6-Minute walking distance
DLCO: Diffusing capacity of the lungs for carbon monoxide
OLDW: OptumLabs® Data Warehouse
ICD-9: International Classification of Diseases, Ninth Edition
ICD-10: International Classification of Diseases, Tenth Edition
HEDIS: Healthcare effectiveness data and information set
T1DM: Type 1 diabetes mellitus
ACE: Angiotensin-converting enzyme
ARB: Angiotensin receptor blockers
SGLT2: Sodium-glucose co-transporter-2
DPP4: Dipeptidyl peptidase-4
GLP-1: Glucagon-like peptide-1
IPTW: Inverse probability treatment weighting
SD: Standard deviation
AMPK: Adenosine monophosphate-activated protein kinase
IGF-1: Insulin-like growth factor
BMP2-PPAR: Bone morphogenetic protein-2-peroxisome proliferator-activated receptor
